# Performance prediction equation for the Valencia Marathon based on time and pacing in the half marathon

**DOI:** 10.3389/fphys.2025.1718298

**Published:** 2026-02-02

**Authors:** Fran Oficial-Casado, Jose Ignacio Priego-Quesada, Pedro Pérez-Soriano

**Affiliations:** Research Group in Sports Biomechanics (GIBD), Department of Physical Education and Sports, University of Valencia, Valencia, Spain

**Keywords:** running, race time, Valencia City of running, training, durability

## Abstract

**Introduction:**

Although pacing is a variable that affects marathon running performance, there is a lack of studies that assessed whether it can improve performance prediction. The aim was to calculate a linear regression model with data such as the half marathon race time, age category, sex and pacing range (difference between the maximum and minimum relative speed of the half marathon) to predict the marathon time. Moreover, the accuracy of the prediction equation obtained was compared with the Daniels’ VDOT.

**Methods:**

A total of 8.261 runners, who participated in both events (Valencia Half Marathon and Marathon) in the same year, for the 2022 and 2023 editions, and ran the half marathon faster than the marathon, were included in the study. Three linear regression models were obtained: a first model with only the half marathon time and sex, a second model adding the age category to these, and a final model adding the pace range to the previous ones. Afterwards, the most accurate and simple model was selected, and its fitting was compared with respect to a model contrasted by the literature, the VDOT.

**Results:**

The introduction of the pace range variable did not improve the model’s prediction, obtaining an explained variance of 85% and an mean absolute error of 5.9%. The overall accuracy of the model obtained was similar to that of the VDOT system, but the models behaved differently depending on the level of runners’ performance.

**Discussion:**

These results allow coaches and runners to establish specific training rhythms to work on the competition pacing.

## 1 Introduction

The participation of recreational runners in endurance events such as the marathon has increased exponentially in recent years (Vitti et al., 2020). The goal of many of these runners is to obtain the best possible race time, and although there is robust evidence about the main physiological determinants of marathon performance (Jones et al., 2021; Joyner et al., 2020), race pacing has recently taken on an important role in the performance of both, elite and recreational runners (Billat et al., 2020; Oficial-Casado et al., 2020; Smyth et al., 2022; Smyth and Muniz-Pumares, 2020). The term pacing refers to the velocity or power distribution during an exercise task or athletic competition (Abbiss and Laursen, 2008). Although a negative pacing profile (consisting in perform the second half of the race at a higher relative speed than the first half) is considered one of the best strategies to get the highest performance (Casado et al., 2021; Díaz et al., 2018), as the level of the runners decreases, running pacing is more heterogeneous, with lower performance in the second half of the race (Oficial-Casado et al., 2022; Smyth, 2021).

While some studies tried to predict marathon performance using data obtained from laboratory tests (e.g., maximum oxygen consumption; VO_2max_), which sometimes is complicated (Keogh et al., 2019; Vandewalle, 2018), others have focused on predicting marathon performance based on demographics or other shorter-distance events (Keogh et al., 2019). In this sense, one of the most popular equations for predicting race performance in long-distance events is the Riegel equation (Riegel, 1981), which takes into account the race time of shorter distances, age group and sex. The validity of this equation is good for distances up to half marathon, but it can be improved in longer distances such as the marathon (Vickers and Vertosick, 2016). Another widely used method to predict long-distance running performance is Daniels’ VDOT (Daniels, 2022), which integrates VO_2max_, the VO_2max_ fraction sustained during a race, and running economy into a single value, allowing for performance comparisons across different distances. One reason for the lower precision of this equation for the marathon could be the effect of durability, which is defined as “the time of onset and the magnitude of any deterioration in physiological profiling characteristics over time during prolonged exercise” (Maunder et al., 2021). Although it has recently been suggested that including the running pacing and the effect of durability could improve the accuracy of performance prediction equations in long-distance events such as the marathon (Jones, 2023; Muñoz-Pérez et al., 2024), there is a lack of evidence on how pacing could improve performance prediction.

The present study aimed to assess the relationship between marathon and half marathon pacing, to propose a performance prediction equation for the Valencia Marathon, considering the race time and pacing of the half Marathon, the age group and the sex of the runners, and to compare the accuracy of the prediction equation obtained with the Daniels’ VDOT. We selected the half-marathon time and pacing to predict marathon time, as it is a common event in marathon preparation and represents a substantial race distance, in which pacing strategy may have a similar effect to that observed in the marathon. This study hypothesized that the marathon performance can be predicted accurately from a simple equation considering the runners’ performance level, the pacing and the race time of a half marathon, with better results than the Daniels’ VDOT.

## 2 Materials and methods

### 2.1 Study design

This is a retrospective study based on the results obtained in the 2022 and 2023 Valencia Half Marathon (https://www.valenciaciudaddelrunning.com/medio/ediciones-anteriores-medio-maraton) and Valencia Marathon (https://www.valenciaciudaddelrunning.com/maraton/resultados-ficha) (first December’s weekend). The Valencia Half Marathon was held 6 weeks before the Marathon (third October’s weekend), forming part of the race organization circuit *Valencia City of Running*. These events are internationally recognized for their fast and flat courses, with minimal elevation gains (69 and 76 m for half and full marathon, respectively) and generally mild weather conditions during this period of the year (15 °C–20 °C and 10 °C–15 °C for half and full marathon, respectively). Number of participants that finished the race was: 21.813 in the marathon of 2022, 26.251 in the marathon of 2023, 17.063 in the half marathon of 2022, and 19.493 in the half marathon of 2023. This method was chosen, aiming to select a large sample of runners of different sexes, categories and performance levels who ran both races in the same year to try to avoid large differences in the runners’ performance.

### 2.2 Database

The study was approved by the Ethics Committee (ref. H1544598666277). The results of the aforementioned races were provided by the organizing entity to the authors. The classifications obtained the following data from the runners: name and surname, age category, sex, splits every 5 km and final race time.

Runners who took part in both races in the same year were selected (n = 8.261, of which 3.817 runners participated in the 2022 edition and 4.444 runners in the 2023 edition). In addition, runners who were missing a split in the classification and those who ran the Half Marathon at a slower pace than the Marathon were discarded, leaving the selectable sample at a total of 7.663 cases.

### 2.3 Data analysis

Participants were categorized by sex, age group (senior, M-35, M-40, M-45, M-50, M-55, M-60, M-65 and M-70) and marathon race time (<2:30, <3:00, <3:30, <4:00, <4:30, <5:00, <5:30 and <6:00 h). The average speed of both tests and the average speed of each split were calculated individually for each runner. The relative speed of each runner’s split was calculated as a percentage of the average speed of the race. The variable pacing range was calculated as the difference between the maximum and minimum relative speed of the half marathon splits (Nikolaidis and Knechtle, 2018; Oficial-Casado et al., 2020).

We used the official VDOT tables from the fourth edition of *Daniels’ Running Formula*, which report, for each VDOT value, the expected race times for several standard distances, including the half marathon and the marathon. For each runner, the official half-marathon finish time was converted from hh:mm:ss to seconds. This value was then matched to the closest half-marathon time listed in the VDOT table, and the marathon time reported in the same row was taken as the predicted marathon performance according to Daniels’ VDOT. This look-up procedure was implemented in RStudio program (version 2024.12.1) using a custom function that, for each observed half-marathon time, returned the marathon time associated with the nearest half-marathon entry in the VDOT table.

### 2.4 Statistical analysis

The statistical analysis was performed with the RStudio program (version 2024.12.1), and the significance limit was established for p < 0.05. First, the correlations between the half-marathon and marathon race times, the half-marathon pace range and the half-marathon mark, and the half-marathon pace range and the marathon mark were analyzed. Afterwards, to verify that the pacing range, obtained from the half marathon data, was related to their finish time in the marathon, the differences in half marathon pacing range between the groups of runners classified by their marathon time were analyzed using an ANOVA with Bonferroni *post hoc*.

The database was then divided into two, taking into account marathon completion time, gender, and age group. Both divisions consisted of 80% of the data to calculate the regression equation, and 20% to test its error. This ratio was chosen because, given the large sample available, it provides an appropriate balance between training and testing sets, which is essential for an unbiased estimation of model performance (Xu and Goodacre, 2018). Three simple linear regressions were performed with the aim of predicting the marathon time. In the first (Model 1) the mark in the half marathon and sex were included. In the second equation (Model 2) the same was done, adding the variable pace range. In the last model (Model 3), the age group variable was added to those already included in the second equation. We also explored potential non-linear relationships using polynomial, exponential and power-law regressions. These models did not improve explained variance or prediction error relative to the linear model, supporting the adequacy of a linear approach for this dataset. The models were adjusted by eliminating the non-significant variables (p > 0.05). After this, the best model was selected based on its simplicity (fewer variables required) and level of variance explained. The variance explained by each predictive variable in the models was calculated by dividing the standardized coefficient of the predictive variable by the sum of all the standardized coefficients of the model, multiplied by the sum of the explained variance (*R* ^2^) of the model.

Following that, a VDOT value was applied to each runner based on their half marathon time and both equations were applied to the testing database, to predict the marathon time and compare it with the real finish time, obtaining the absolute error percentage between them. A three-factor ANOVA analysis was performed to observe differences between the models, the time category, and the effect of the model on the different categories. Bonferroni’s *post hoc* test was applied and Cohen’s effect sizes (ES) (small (0.2), medium (0.5) and large (0.8)) were calculated (Cohen, 1988) for pairwise comparisons of the errors of both models and the different categories. To assess the agreement and consistency of the different models in the different marathon time categories, the intraclass correlation coefficient (ICC) (Based on a 2-way random-effects model (“2,1”) (Shrout and Fleiss, 1979), between the models’ estimated time in the marathon time categories and the actual mark was calculated. The following classification of ICC values was used (Weir, 2005): values from 1.00 to 0.81 (excellent reproducibility), from 0.80 to 0.61 (very good), from 0.60 to 0.41 (good), from 0.40 to 0.21 (reasonable) and from 0.20 to 0.00 (poor).

## 3 Results

### 3.1 Relationship between marathon and marathon race time

**FIGURE 1:**
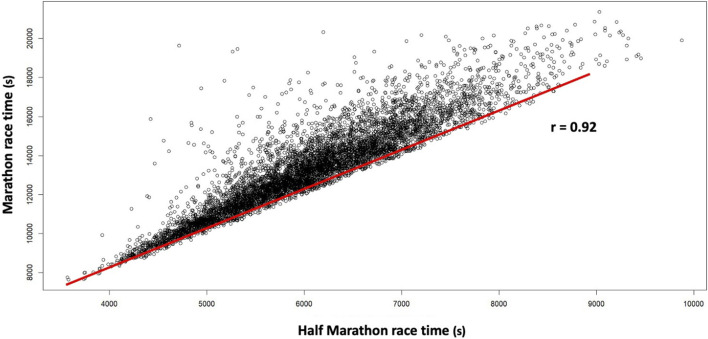
Simple linear correlation between the Valencia Half Marathon and Marathon race times in 2022 and 2023.

The Valencia Half Marathon race time showed a strong positive correlation (p < 0.001; r = 0.92) with the Valencia Marathon race time (Figure 1). On the other hand, moderate positive correlations were also observed between the pacing range of the Valencia Half Marathon and the Half Marathon (p < 0.001; r = 0.39) and Valencia Marathon (p < 0.001; r = 0.39) race time.

### 3.2 Relationship of half marathon pacing range and marathon performance

**FIGURE 2:**
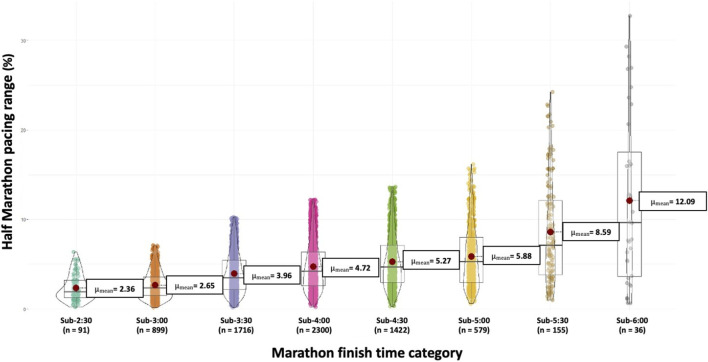
Pacing range (difference between the maximum and minimum relative speed of the half marathon splits) of the runners classified by their marathon finish time.

The pacing range of the half marathon was higher as progress was made in the marathon finish time category (Figure 2), with the sub-2:30 h category being lower than all categories from the sub-3:30 h (p < 0.01), and the sub-3:00 h category being lower than all categories from the sub-4:00 h (p < 0.01), The sub-3:30 h was lower than all categories from the sub-4:30 h (p < 0.01), and so on.

### 3.3 Regression models obtained

The explained variance obtained from the three models proposed was similar (*R* ^2^ = 0.85) (Table 1), even though in both (Model 2 and Model 3), the pacing range of the Half Marathon provided significant differences (p < 0.001). In Model 3, some significant differences were also observed when considering the age group in certain categories (U-20 (p < 0.001), U-23 (p < 0.01) and Senior (p < 0.001)) (Table 1). The average ICC between estimated and real race time of Models 1 and 2 was excellent (ICC = 0.92). The ICC coefficients in the different time categories were consistent in both models: very good reproducibility in sub-3:00 and sub-3:30 (0.70 and 0.60 respectively in both), good in sub-4:00 (0.47 and 0.46 respectively), reasonable in the sub-4:30 h category (0.35 in both), and poor in the slower categories (ICC Model 1 and two sub-5:00, sub-5:30 and sub-6:00 = 0.02–0.08). Based on its simplicity (fewer variables required) and effectiveness, Model 1 was chosen for the following analyses.

**TABLE 1:** Summary of the regression models obtained to predict marathon time in seconds.

Model	Variance explained by the model	Predictor variables		Coefficients	IC95%		Predictor-explained variance (%)
Model 1	*R* ^2^ = 0.85	Intercept		−701.85	−883.06	−520.64	-
		Half marathon time (s)***		2.28	2.25	2.3	80
		Gender (male)***		329.59	264.01	395.16	5
Model 2	*R* ^2^ = 0.85	Intercept		−622.97	−808.06	−437.88	-
		Half marathon time (s)***		2.25	2.22	2.28	79
		Gender (male)***		313.25	247.26	379.23	4
		Half marathon pace range***		16.12	8.19	24.06	2
Model 3	*R* ^2^ = 0.85	Intercept		−738.97	−933.84	−544.11	-
		Half marathon time (s)***		2.27	2.24	2.3	76
		Gender (male)***		339.7	273.39	406.02	4
		Half marathon pace range***		14.07	6.14	22	2
		Age group	Sub-20***	1194.23	506.61	1881.85	3
			Sub-23**	380.44	108.78	652.1	
			Senior***	200.61	122.44	278.77	
			M-35	−46.93	−121.98	28.12	
			M-40	−34.52	−110.04	41	
			M-45	−29.18	−111.23	52.87	
			M-50	38.45	−68.2	145.1	
			M-55	16.8	−140.2	173.79	
			M-60	49.33	−212.1	310.77	
			M-65	−268.03	−738.22	202.16	
			M-70	1119.96	−560.67	2600.58	

***p < 0.001; **p < 0.01; *p < 0.05.

Categorization of age groups according to the regulations of the World Athletics and the RFEA (available on the race’s website: https://www.valenciaciudaddelrunning.com/maraton/reglamento-42k-2024/#seccion4): U-20: 18 and 19 years old; U-23: 20, 21 and 22 years old; Senior: 23–34 years; M-35: 35–39 years old; M-40: 40–44 years; M-45: 45–49 years old; M-50: 50–54 years; M-55: 55–59 years old; M-60: 60–64 years; M65: 65–69 years; M70: 70–74 years.

The marathon race time prediction equation obtained from Model 1, but in minutes, was as follows:

$$Valencia\;Marathon\;race\;time\;\left(men\right)\;\,\left(\min\right)=-6.21+2.28*Valencia\;Half\;Marathon\;race\;time\;\left(\min\right)$$

$$Valencia\;Marathon\;race\;time\;\left(women\right)\;\,\left(\min\right)=-11.70+2.28*Valencia\;Half\;Marathon\;race\;time\;\left(\min\right)$$

### 3.4 Comparing Model 1 to VDOT

The VDOT average ICC was also excellent (ICC = 0.83). The results of the ANOVA analysis showed differences (p = 0.02) between the predictions of both models and between the predictions of each model in the different time categories (p = 0.04).

**FIGURE 3:**
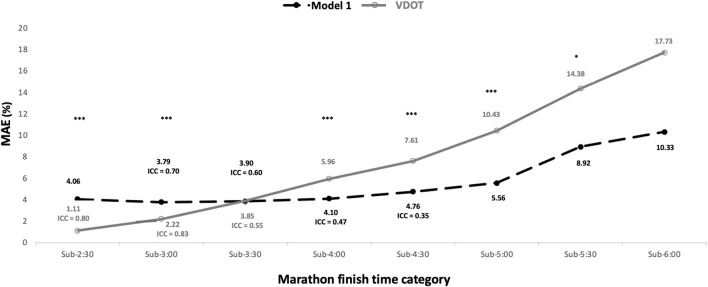
Evolution of the mean absolute error (MAE) (%) of Model 1 and VDOT as a function of performance (time category) in the marathon. For ease of understanding, error bars are not displayed. The differences in precision (MAE) between the models are indicated by * (p < 0.05) and *** (p < 0.001). The ICCs indicated for each model correspond to ICCs >0.20 and p < 0.05. In grey, values corresponding to the VDOT, and in black, values of Model 1.

Model 1 obtained an average mean absolute error (MAE) of 5.67%, while that of the VDOT was 7.92%. The MAE of the models showed differences in all time categories with moderate to large effect sizes (sub-2:30, p < 0.001 and ES 2.12; sub-3:00, p < 0.001 and ES 0.81; sub-4:00, p < 0.0001 and ES 0.49; sub-4:30, p < 0.0001 and ES 0.61; sub-5:00, p < 0.001 and ES 0.81; and sub-5:30, p < 0.05 and ES = 0.6), except in the sub-3:30 and sub-6:00 categories (Table 2). Model 1 was less accurate than VDOT in the highest performance time categories (sub-2:30 and sub-3:00), while it progressively improved its accuracy with respect to VDOT from the sub-4:00 category (Table 2; Figure 3).

**TABLE 2:** Mean absolute error (MAE) (%) of Model 1 and VDOT in the different marathon time categories.

Time category	Model 1				VDOT				Differences	
	MAE	95%CI		SD ±	MAE	95%CI		SD ±	p	ES
	(%)	Lower	Upper	(%)	(%)	Lower	Upper	(%)		
Sub-2:30	**4.06**	3.10	5.02	1.73	**1.11**	0.54	1.69	1.04	**< 0.001**	**2.12**
Sub-3:00	**3.79**	3.46	4.12	2.17	**2.22**	1.97	2.47	1.63	**< 0.001**	**0.82**
Sub-3:30	3.85	3.55	4.15	2.85	3.90	3.56	4.24	3.24	0.83	-
Sub-4:00	**4.10**	3.79	4.41	3.25	5.96	5.54	6.38	4.42	**< 0.001**	**0.49**
Sub-4:30	**4.76**	4.29	5.23	4.03	7.61	6.99	8.23	5.31	**< 0.001**	**0.61**
Sub-5:00	**5.56**	4.38	6.74	5.68	10.43	9.10	11.77	6.41	**< 0.001**	**0.81**
Sub-5:30	**8.92**	5.67	12.18	9.32	14.38	11.27	17.48	8.90	**0.049**	**0.60**
Sub-6:00	10.33	2.44	18.21	7.52	17.73	11.82	23.64	5.63	0.25	-

Standard deviation (SD), 95% confidence interval (95%CI), p-values with Bonferroni adjustment and effect size (ES) of the differences were shown. Differences (p < 0.05) were highlighted in bold letters.

## 4 Discussion

Several marathon-performance prediction equations have been proposed, often with a lot of heterogeneity of variables, based on the runners’ characteristics and their performance (Alvero-Cruz et al., 2020; Keogh et al., 2019; Muñoz-Pérez et al., 2024), which can make it difficult to implement (Keogh et al., 2019; Vandewalle, 2018). The main objective of this study was to obtain a prediction of the marathon race time through an equation that included easily accessible data for all runners, such as age group, half-marathon race time, sex, and the variable half-marathon pacing range. The results show how, with only the half marathon finish time and sex, it was possible to explain 85% of the variance in marathon performance, with an excellent ICC (0.92) and a MAE of 5.7% in a large and heterogeneous sample. These results are positive considering the heterogeneity of the values obtained by equations from previous studies, in which other variables of more difficult access were obtained through laboratory, field tests and training data (Alvero-Cruz et al., 2020; Keogh et al., 2019). In other predictive studies with large databases, the variance explained was similar to that of the models proposed in this study, both by applying a critical power model (Smyth et al., 2022; Smyth and Muniz-Pumares, 2020), artificial intelligence (Lerebourg et al., 2022) or a simple linear regression model with similar predictors (Muñoz-Pérez et al., 2024).

When adding the pacing range variable in Models 2 and 3, the explained variance did not increase with respect to Model 1, and the ICC behaved consistently in both models. This likely reflects that half marathon performance already captures much of the influence of effort distribution on outcome, consistent with prior work showing pacing’s impact on half-marathon performance (Nikolaidis et al., 2019; Ristanović et al., 2023). The results of this study are in line with those of (Muñoz-Pérez et al., 2024), where half marathon performance explained much of the variance of the regression model to predict marathon performance, and adding variables intended to represent marathon pacing or group behavior yielded minimal gains. Similarly, when taking into account the decoupling between internal and external load (Smyth et al., 2022), observed that, although this variable significantly improved the model’s prediction, most of the marathon performance was explained by runners’ critical speed. Moreover, most of our sample completed the half marathon under 2 h, duration that may be insufficient to elicit meaningful decrements in durability (Clark et al., 2018). Therefore, extrapolating durability effects from efforts under 2 h to marathon durations may underrepresent runners’ resilience (Jones, 2023).

Performance based on age group was only different in the under-34 age groups, probably because most of the fastest runners (<2 h 30 min) are included in this age group. Different studies agree that maximum performance decreases, or is affected, approximately from the age range between 35 and 40 years old (Reusser et al., 2021; Smyth and Muniz-Pumares, 2020; Zavorsky et al., 2017). However, especially when analyzing recreational runners, the results of other studies also found trivial interactions between age and performance (Muñoz-Pérez et al., 2024; Nikolaidis and Knechtle, 2017). Considering that much of our sample comprised recreational runners with times above 3 h, many far from their age-group bests, age may be less limiting at these performance levels, whereas experience and training load likely play larger roles (Reusser et al., 2021; Smyth, 2021; Vickers and Vertosick, 2016).

When comparing the Model 1 and the VDOT, both models showed excellent agreement between the estimated and actual race time (ICC >0.8). However, the accuracy of the models behaved differently depending on the runners’ performance level: the VDOT system was more accurate in the fastest categories (sub-2:30 h and sub-3:30 h), while Model 1 was more stable in its accuracy throughout the different categories and improved over the VDOT from sub-4:00 h (and in other slower categories). Something similar happened in other studies that compared groups of runners of different levels with the VDOT system (Scudamore et al., 2017). It is suggested that this difference in model dynamics may be due to bias due to the origin of the models. The VDOT is based on a power-law based on trained runners with a higher level of performance, while Model 1 is based especially on the behavior of the most crowded categories (between sub-3:30 and sub-5:00). Although Model 1 fits better in the most crowded categories, the results show how accuracy decreases especially from the sub-4:30 h category, where performance is more heterogeneous, possibly due to the effect of greater variability in relative intensity and, consequently, in running pacing (Billat et al., 2019; Smyth, 2021; Smyth et al., 2022; Smyth and Muniz-Pumares, 2020). In this sense, it is recommended that, in the choice of one predictive model or another, the parsimony of the model (Keogh et al., 2019; Vandewalle, 2018; Zinoubi et al., 2017) and the runner’s profile (García-Manso et al., 2012; Mulligan et al., 2018) should be taken into account. When the race time is available in different tests of durations similar to the one aimed to predict, generally the models based on power-laws fit better (Vandewalle, 2018; Zinoubi et al., 2017). However, in long events such as the marathon, it is difficult to have similar performances close to the target event, and in turn, in these cases, the fact of having a prediction to establish the pacing strategy becomes more important (Coquart, 2023). Regarding this, easy-to-implement models such as the one proposed in this study can be interesting.

The mechanisms of inter-individual variability in the resilience capacity of runners are still unknown (Jones, 2023; Jones and Kirby, 2025), so the introduction of variables or tests that can reflect the runners’ resilience is suggested to improve predictive models (Jones and Kirby, 2025). In addition, in future studies it would be interesting to assess the prediction of marathon performance through specific tests of longer duration or load (considering the relationship between duration and intensity) that can better reflect the individual durability of runners and whose results are easily applicable in a predictive model.

These results are interesting for runners and coaches in setting realistic race goals, planning appropriate pacing strategies, and guiding training intensity in the weeks leading up to the marathon. However, it is important to note that the equation provided is specific to the Valencia Marathon and may not be applicable to other events of different characteristics (Oficial-Casado et al., 2020). It would be convenient to explore the behavior of this predictive model in other races with similar characteristics. Moreover, the database provided by the race organizers did not include relevant variables such as training volume, weekly mileage, runners’ training history, or years of endurance running practice. These factors are known to influence endurance performance and could potentially improve the predictive capacity of future models. Consequently, future research should incorporate these variables and test the model to evaluate its broader applicability.

In conclusion, a simple, accurate and valid performance prediction equation was obtained in a large and heterogeneous sample of runners. The introduction of the variables half-marathon pace range and age group in the regression model did not improve the prediction of the marathon finish time. The behavior of the model obtained was consistent in relation to VDOT system, although at certain performance levels, its accuracy was different, so it is recommended that runners choose the model that best suits their individual characteristics.

## Data Availability

The datasets presented in this study can be found in online repositories. The names of the repository/repositories and accession number(s) can be found below: Mendeley repository (doi: 10.17632/fbtbc38fhr.1).
